# British Medicinal Plants

**Published:** 1891-02

**Authors:** Alfred Heath


### BRITISH MEDICINAL PLANTS.

ALFRED HEATH, M. D., F. L. S.

### ORDER 6.—CRUCIFERÆ (CONTINUED.)

*Diplotaxis tennifolia* (Wall Mustard, Wall Rocket).—Found on old walls. This plant is said to have followed the Romans or to have been introduced by them; if so, it is now thoroughly acclimatized and may be found plentifully in many parts of the country, especially on heaps of rubbish and on the walls of great towns in the south and southwest and east of England. It is a *perennial* plant with leafy stem, and a very disagreeable odor. The Diplotaxis (of which there are only two examples found in this country) belong to the tribe Brassiccæ, but is distinguished from Brassica and Sinapis by the *double* rows of seeds in the pod. There is no proving of this plant, but its virtues are probably similar to the mustards.

*Diplotaxis muralis* (Rocket).—This plant is very similar to the last mentioned, but is an *annual* and much smaller, with no leaves on the stem, but it has a rosette of leaves at the base of the stem. Its properties are probably like the D. tennifolia, as it smells just the same.

*Thlaspi arvense* (Penny Cress. Field Penny Cress. Trade Mustard).—Found in fields and roadsides. The seeds of this plant have an acrid, biting taste approaching to that of common mustard. They have an unpleasant flavor somewhat like garlic or onions, they are also bitter. This was at one time a Pharmacopœia plant, and was considered diuretic, provoking urine, and helping dropsy, gout, sciatica, and forwarding the menstrual functions. The seeds are the part used, and the country people give them to destroy worms and with good effect. They are also given in obstruction of the viscera, in rheumatism, and jaundice with success. They operate moderately on the urinary organs in small doses, in larger they purge briskly and in still greater quantities cause vomiting. There is at present, I believe, no proving.

*Iberis amara, Lepidium Iberis* (Bitter Candy Tuft. Sciatic cress).—So named from Iberia (Spain), where many kinds of Iberis are found. Found in chalky fields in the south and east of England. It has been used with considerable success as a cure for sciatica. It is said to be antiscorbutic, antiseptic, and stomachic. There is a proving in Hering's *Guiding Symptoms.* Among many other things it produces various disturbances of the digestive organs, as well as of the heart and lungs.

*Capsella Bursa-pastoris. Thlaspi Bursa-pastoris* (The Shepherd's Purse, a common weed).—This plant is said to have most extraordinary virtues, but is comparatively little used in medicine. It has a great reputation as a healer of outward and inward wounds; hemorrhages, spitting and voiding of blood; in jaundice, inflammation, erysipelas, pains and noises in the ears, and wounds on the head. It has been successful in treating passive metrorrhagia with too copious and frequent menses, as also in delayed menses caused by inertia of the uterus.

*Armoracia Sativa. Cochlearia Armoracia* (The Common Horse-radish).—Common in our gardens, but not a native and never seeds here. Called armoracia because it was cultivated abundantly in Armorica. This plant is well known at our tables, and although there is not the *least* similarity between them, the poisonous root of aconite has been used in mistake for the horse-radish by ignorant people, and death has been caused. The horse-radish has a long, stout white root about a foot long, and affects the organs of taste and smell with a quick, penetrating pungency, whereas the aconite root is of a darker color, tapering from the top, and not more than three or four inches in length, and has numerous fibrous roots as well; it is almost tasteless, and the numbing, tingling sensation it produces on the tongue and throat is not felt for five or ten minutes after it has been taken into the mouth. The activity of horse-radish is largely owing to an acrid substance similar to that found in black mustard. Its virtues are much impaired by drying, and the tincture should always be made from the fresh root. In allopathy, armoracia has been given as a remedy in rheumatism and palsy; for hoarseness; as a stomach stimulant; to promote digestion; also as a powerful *diuretic*, and with success in dropsy.

Hering's *Guiding Symptoms*, vol. I, gives a good proving of the drug. It produces on the healthy, *and has frequently cured in the diseased*, the following affections: "*Rheumatism of joints, which is better from motion and worse from rest. Loss of voice: whispering. Aphonia, with blood-spitting. Hoarseness and roughness of throat*; it is used by singers to clear the throat. *Violent cramps in stomach, beginning toward morning, continually increasing, driving to despair; cramp in stomach after taking cold. Greatly increased secretion of urine. Dropsy, with albuminuria, after pneumonia; beginning of enteritis; beginning of pleuritis.*"

### Order 9.—Violaceæ.

*Viola-odorata* (Sweet Violet, Wood Violet).—Every one in the country knows where to find the violet, and I suppose there is no one who does not love its beautiful and delicate perfume, reminding one of the spring and green fields. Well may Venus claim this plant. As a drug the violet possesses very cooling properties, and has been used in heated conditions of the body, in inflammation of the eyes, hot swellings in different parts, pain in the head from want of sleep, with heat; to help suppuration in pleurisy, and affections of the lungs and chest; hoarseness; in affections of the liver; in the hot stage of ague, piles, etc., and it is said to remove stone in the bladder.

*Viola-tricolor* (Heart's Ease, Pansy).—A very troublesome weed on cultivated grounds, corn-fields, etc., in England. Many old writers on *Materia Medica* represent this plant as a powerful medicine in epilepsy, asthma, ulcers, scabies, and other cutaneous complaints especially, as it has been recommended as a remedy for *crusta lactea*, convulsions of children, in pleurisy and other chest troubles. The heart's ease was at one time reckoned among the *magic herbs*. There is a proving of this plant in Dr. T. F. Allen's hand-book of *Materia Medica*. Amongst other things it produced drawing and twitching in the limbs, twitching of hands and closing of the thumbs, twitching of pectoral muscles, sticking in the ribs on left side during inspiration and expiration, cutting in chest on movement, oppression in region of heart, with twitches, eruptions on the face and behind ears, with burning itching, worse at night; thick hard scabs formed, crack here and there, discharging tenacious yellow pus, which hardened into a substance like gum. Nettle rash. Itching, twitches, cutting and crawling, itching of nose, scapula, inner part of thigh, anterior part above knee, on ball of big toe, between scrotum and thighs.

### Order 10.—Droseraceæ.

*Drosera rotundifolia* (common name, Sun-dew, Round Leaved Sundus).—Found in boggy places. The tincture should always be prepared from the green plant in flower, care being taken to exclude the other forms of drosera found growing with it—*i. e.*, D. intermedia, D. longifolia. The action of the two last named may be similar, but we do not know. (The Scotch shepherds use drosera as a remedy for hæmaturia in cows, but D. rotundifolia does not appear to produce this form of hemorrhage. Perhaps one of the other forms of drosera would produce this symptom, and the cure of the hæmaturia in cows by the shepherds may be on account of one of the other forms producing this symptom. (See an amusing controversy in *Homœopathic World*, beginning April, 1883, page 150, bottom lines.) Beside the other forms of drosera that are often gathered in error, there is in one pound of *apparently* clean looking fresh plant, when carefully sorted, quite a quarter of a pound of pieces of grass, moss, and other extraneous matter picked out. In the dried drosera it is quite impossible to separate these things from the plant, so that the tincture from dried drosera is never pure.

This well-known and elegant little plant was at one time used to remove warts and corns. It is so acrid that when applied to the skin it has been known to produce ulceration. Before any proving of the drug was made it was esteemed as a remedy in *asthma and coughs.* It produces fatal coughing and delirium in sheep who eat it. In Homœopathy it is a prominent and common remedy in whooping-cough and various kinds of spasmodic cough. There is a good proving of the drug, showing its action on healthy persons. The following are some of the symptoms *produced*, and they are cured by drosera when they arise from natural causes:

Bleeding of the nose; frequent sneezing, with or without coryza; profuse fluent coryza, particularly in the morning; voice hoarse, deep, requires exertion to speak, husky, hollow, toneless; constriction of the larynx when talking; sensation as of a feather in the larynx, exciting to cough; chest and throat; symptoms worse from talking and singing; desire to support the larynx when swallowing or coughing; oppression of the breathing; periodical paroxysms of whooping-cough in such frequent successions that the breath can scarcely be taken; worse in the evening on lying down and at night, with drawing in of the abdomen, with vomiting of water, mucus, and food; bleeding from the nose and mouth; aggravation of the cough, by warmth, drinking, tobacco smoke, laughing, singing, weeping; after lying down, after midnight, or in the morning, and many other chest symptoms. It produces aversion to pork.

Orders 11, 12, and 13 contain no English plants that are at present used in Homœopathy.

